# Conceptual framework for creative dance-based practice in cerebral palsy rehabilitation: mini review

**DOI:** 10.3389/fresc.2025.1758651

**Published:** 2026-01-16

**Authors:** Hee Joung Joung

**Affiliations:** 1 Sport Science Laboratory, Changwon National University, Gyeongnam, Republic of Korea; 2 Department of Orthopedic Surgery, Seoul National University Bundang Hospital, Gyeonggi, Republic of Korea

**Keywords:** cerebral palsy, creative dance, Laban-based movement, rehabilitation, task-specific approach

## Abstract

In recent years, shifting paradigms in cerebral palsy (CP) rehabilitation have highlighted approaches that promote autonomous movement through active participation, including goal-directed training, task-specific training, context-focused therapy, and constraint-induced movement therapy. Dance has increasingly been recognized in neurorehabilitation as a promising intervention capable of supporting physical, cognitive, and social functioning. Among various dance practices, this mini review focuses on the conceptual framework of creative dance. Creative dance centers on the process of generating movement, which aligns with contemporary paradigms in cerebral palsy rehabilitation. Additionally, because creative dance values the discovery and development of new and unique movements, it provides a setting in which movements, whether with or without disabilities, are respected and accepted as having equal value. These values play a crucial role in fostering a sense of achievement, interest, motivation, and sustained engagement, all of which are emphasized in rehabilitation for individuals with cerebral palsy. However, despite its potential, creative dance remains in the early stages of development as a rehabilitation modality. Establishing a solid evidence base will require a clearer understanding of the movement methodology underlying creative dance. In this mini review, I examine the movement methodology embedded in creative dance–based practice (CBP) and propose a conceptual framework to support its implementation within rehabilitation contexts for individuals with CP.

## 1 Introduction

Recent theoretical and empirical studies have begun to position dance as a promising multimodal intervention in neurorehabilitation, capable of simultaneously engaging motor, cognitive, emotional, and social domains across neurological conditions such as Parkinson's disease (PD), stroke, dementia, and neuromotor disorders. An expanding body of research in PD, in particular, has demonstrated meaningful improvements in gait, balance, non-motor symptoms, and quality of life, and dance programs have increasingly transitioned from clinical settings into community, public health, and patient-driven contexts (1).

Emerging research in individuals with cerebral palsy (CP) has similarly suggested that dance-based interventions may offer motor as well as potential cognitive and psychosocial benefits (2–7). Among various dance styles, creative dance (CD) is of particular interest because it is process-oriented and emphasizes movement exploration, improvisation, sensory awareness, self-directed adaptation, and the discovery of diverse movement solutions (8, 9). This orientation differs from traditional structured dance forms, which require learners to reproduce predetermined steps and choreographed movement patterns. These characteristics align closely with the aims of contemporary intervention for CP, which does not primarily aim to promote the learning of “normal movement patterns,” but instead seeks to promote movement control, adaptability, functional performance, self-generated active movements, participation, sustained participation, social interaction, and independence (10–12).

Although CD has been widely used in educational settings, its application in CP rehabilitation remains at an early stage. This may be partly due to the inherently open-ended and non-prescriptive nature of creative dance, which can make it challenging to standardize or structure within traditional rehabilitation paradigms. Dance-based interventions are inherently difficult to reduce to rigid, prescriptive protocols, as they rely on improvisation, relational interaction, and real-time adaptation to participants' physical and psychosocial states (13). These characteristics are particularly pronounced in rehabilitation contexts, where individual variability and moment-to-moment clinical decision-making are integral to the intervention process. Importantly, however, difficulty in standardization does not preclude methodological clarity. As demonstrated by Beaudry et al. (13), who described an adapted dance intervention for subacute post-stroke rehabilitation, explicitly articulating both *what* the intervention consisted of and *how* it was delivered provides a valuable framework for enhancing transparency, reproducibility, and clinical translation. In line with this perspective, López-Ortiz et al. (2) and Duarte Machado et al. (3) emphasize that the advancement of dance as a rehabilitation intervention for individuals with CP requires detailed descriptions of interventions fidelity and replication procedures, as well as the development of theoretical models that can guide program design, implementation, and interpretation.

Therefore, the present review aims to explore a conceptual direction for creative dance-based practice (CBP) in CP rehabilitation. By articulating the underlying principles that may guide such approaches, this review seeks to inform future research and support the advancement of dance as a rigorous and meaningful rehabilitation modality for individuals with CP.

## 2 Paradigm shifts in cerebral palsy rehabilitation: toward functional, active, and adaptive motor practice

Rehabilitation for individuals with CP has undergone a notable conceptual shift in recent decades. At the center of this shift is an increased emphasis on functional performance and improvements in functional carryover, leading to a growing use of evidence-supported approaches that promote child-centered active engagement, self-generated and adaptive movement, and task or environmental modification to support functional success, rather than relying solely on traditional practices focused on movement normalization or therapist-led passive facilitation.

In line with this paradigm shift, previous studies (10, 14–16) highlight task-specific training, goal-directed training, context-focused therapy, and environmental enrichment as evidence-supported interventions that improve motor performance, activity participation, real-life functional outcomes, and self-initiated movement. Notably, these approaches are underpinned by principles aligned with dynamic systems theory (DST).

DST posits that motor behavior and development emerge from the dynamic interaction among the person, the task, and the environment during the performance of functional activities (17). From this perspective, motor actions are not produced by executing predetermined movement templates; rather, they arise through self-organization, as individuals explore movement possibilities, negotiate task demands, and adapt to environmental constraints. Within this framework, the role of intervention shifts from correcting “abnormal” movement patterns to supporting the child in discovering functional, feasible, and task-specific motor solutions**.** Fetters (18) emphasized that intervention should facilitate the child's ability to explore the movement environment, adapt to changing contextual demands, and select functional solutions when confronted with new task requirements. This theoretical lens provides a compelling rationale for approaches that promote active problem-solving, exploratory movement, adaptability, and variability—elements that are essential for functional motor learning and that likewise underpin the core principles of Creative Dance-Based Practice (CBP). Thus, the central question underlying this paradigm shift is not “what” movement children should perform, but “how” they can regulate, generate, and adapt their own movements within the constraints of tasks, environments, and personal characteristics.

## 3 A conceptual framework for creative dance-based practice as an intervention for cerebral palsy

### 3.1 Creative dance-based practice (CBP) as a movement methodology

CBP positions participants not as imitators of predetermined patterns but as active generators, organizers, and modifiers of their own movement responses. Within dance education, creative dance has long been described as a form of movement-based problem solving, in which individuals explore potential solutions to movement challenges through bodily inquiry and experimentation (8, 9). From this perspective, creative dance facilitates the discovery of new ways of moving by encouraging participants to actively generate, vary, and combine fundamental movement elements.

In this framework, movers are regarded as “movement thinkers”—individuals who search for kinesthetic solutions and express them through self-selected strategies rather than reproducing a single prescribed form. This conceptualization aligns with contemporary rehabilitation paradigms for individuals with CP, which emphasize self-initiated action, goal-directed practice, task-specific repetition, adaptive movement, and meaningful engagement. Although CBP originates from artistic and educational traditions, its learning mechanisms share key learning principles with modern, evidence-supported motor interventions.

### 3.2 Laban movement analysis (LMA) -based movement framework as generative variables for learning

The movement structure of CBP is grounded in the LMA framework, which organizes movement into four fundamental elements. The movement variability framework is presented in Table 1 and refers to concepts from the book *Creative Dance for All Ages* (8) and studies by Joung et al. (7, 19).

**Table 1:** Movement variation framework.

Movement variability = body × f(x) where f represents the modulation of movement through spatial, temporal, and dynamic parameters.	
Elements	Components
Body What we move	Body part: head, arm, hand, torso, pelvis, leg, foot et al Partial-body action (isolated movement) and whole-body action (integrated movement) Locomotor actions: walking, running, hopping, skipping, galloping, chasing, fleeing, dodging Manipulative actions: throwing, catching, kicking, punting, dribbling, volleying Non-manipulative actions: turning, twisting, rolling, balancing, transferring weight, jumping and landing, stretching, curling
Space Where the body moves	Level: low middle high Extension: large/small, far/near Direction: up/down, forward/backward, right/left, clockwise/counterclockwise Pathways: straight, curved, zigzag,
Time When the body moves	Tempo: slow fast Rhythm: pattern
Force How the body moves	Energy: relaxed tense, light strong

The core idea of the movement variability framework in CBP is to position the body as the constant and to generate diverse movements through the combinations of movement elements, including space, time, and force (8, 9, 20). Within this framework, movement is not predefined or fixed; rather, it emerges through the learner's active selection, exploration, and recombination of movement elements. By treating the body as a constant and the movement elements as adaptable and self-directed variables, CBP operationalizes the idea that “movement variability=body×f(x).” In this formulation, the body functions as the stable resource, while *f(x)* represents the learner's adaptable and voluntarily manipulated spatial, temporal, and force-related parameters. As learners modulate these parameters, they continually adjust their motor strategies, discover new coordination patterns, and refine their expressive capacities. In this way, the framework may serve as a fundamental guideline for enhancing movement learning in individuals with CP, potentially supporting adaptable and self-directed movement.

For example, in a walking task (body part: legs) (9, 19), movement diversity can be expanded by integrating space aspects, such as direction and pathways—walking forward, backward, sideways, linear, zig-zag, or curved. Additional time aspects (slow to fast or stop and move) may be layered onto this task, prompting actions such as walking quickly forward, walking slowly backward, or changing direction at a moderate pace. Similarly, in a “drawing shapes with body parts” (7, 8) task, participants might draw circles with their shoulders or create straight lines with their torso. Through such activities, CBP encourages participants to explore and create a wide range of movements using their own bodies. Two randomized controlled studies demonstrated that movement-based interventions grounded in Bartenieff, Feldenkrais, Horton, Graham, and Laban methods, which were focused on enhancing kinesthetic perception of one's own body movement, were associated with improvements in functional independence (4) and lower-limb range of motion (5), compared with conventional physical therapy in individuals with CP classified as GMFCS levels IV–V, suggesting the potential relevance of exploratory movement approaches for individuals with severe motor impairment. This approach reflects a child-focused perspective by developing movement from the individual's current bodily capacities instead of aiming for what is deemed “normal” or “right.”

This perspective is broadly aligned with contemporary rehabilitation interventions in CP—such as functional therapy (16, 21), child-focused and context-focused interventions (22), activity-focused and goal-directed therapy (23), and task-specific training (24, 25)—which emphasize functional performance rather than normalization and seek to enhance children's motor abilities by adjusting task or environmental constraints. In a cluster randomized trial, Law et al. (16) found that children receiving a context-focused, activity-oriented intervention—where therapists modified task or environmental constraints rather than attempting to remediate impairments—demonstrated significant improvements over time on the PEDI Functional Skills Scale and Caregiver Assistance Scales. These findings suggest that altering task and environmental conditions can facilitate functional gains, reflecting the principle that functional performance emerges from the child's exploration of variable movement solutions. In a randomized controlled trial, Rafique et al. (25) demonstrated that children receiving task-oriented training—consisting of clearly defined functional tasks such as unsupported standing with multidirectional reaching, sit-to-stand transitions from the floor, sidestepping, stair climbing, straight-line and tandem walking, inclined walking, walking while carrying a cup of water to support dual-task control, and catching and throwing a ball for dynamic balance—showed significantly greater improvements in walking performance, as evidenced by reduced TUG and FWT times after six weeks. These tasks were progressed by manipulating repetitions, speed, and task transitions, effectively altering the constraints that shape movement. Such improvements highlight how task-specific practice, grounded in variability and constraint modulation, enables children to discover new coordination patterns and adopt more efficient movement strategies—core principles consistent with the proposed movement variability framework.

### 3.3 Creative dance-based practice tasks

Movement learning in CBP is enacted through tasks that incorporate functional actions commonly addressed in CP rehabilitation—locomotion (walking, running, changing directions), object manipulation (reaching, grasping, throwing), and postural transitions. As with the interventions grounded in DST described above, CBP engages participants in active, self-initiated practice of meaningful tasks under developmentally appropriate constraints. Within this framework, movement learning may occur through repeated engagement in trial-and-error processes, as individuals explore a range of possible and creative movement solutions that their own bodies can generate to accomplish a given task.

CBP expands beyond task-specific training by embedding movement tasks within imagined scenarios, narrative frames, and expressive choice-making, thereby enlarging the learner's motor solution space. Table 2 summarizes CBP tasks reported in previous empirical studies that demonstrated improvements in motor function among adolescents and adults with CP. As empirical studies on CBP in individuals with CP that provide detailed task descriptions remain limited (3), Table 2 is therefore restricted to the available studies by Joung et al. (7, 19), supplemented by task descriptions drawn from the book *Creative Dance for All Ages* (8). For example, a walking task may be situated within a narrative such as “moving through a crowded street,” “walking in outer space,” or “moving as if one were an alien.” These playful yet structured contexts stimulate exploration of varied speeds, directions, weight shifts, and dynamic balance strategies (7, 8). Similarly, manipulative tasks such as reaching or throwing can be enriched through imaginative framings—for instance, the “invisible ball” task, in which children imagine the ball's weight, texture, or size and adjust their motions accordingly (19). These scenarios naturally elicit functional variation, motor planning, sensory imagination, and adaptive control, expanding the repertoire of motor solutions without imposing the pressure to “perform correctly.” This is similar to the tasks used in Rafique et al. (25), but rather than training discrete actions such as walking or reaching while walking, CBP situates the activity within an imaginative context—such as playing with an invisible ball—which further amplifies exploratory movement and broadens the range of adaptive motor responses.

**Table 2:** Tasks used in creative dance-based practice for individual with cerebral palsy.

Task	Description	Study
Drawing shapes with body parts	• Participants draw shapes (e.g., their name, signature, or geometric figures) using different body parts. • Single body part: Move using one body part only (e.g., drawing a circle with the shoulder, writing a name with the hip). • Two body parts at the same time: Move two body parts simultaneously (e.g., head × shoulder, shoulder × pelvis, pelvis × knee).	Joung et al. (7), Gilbert (8)
Random walking	• Participants walked around the classroom in a scattered formation, using their own choice of movement elements (e.g., direction, timing, pathways, shapes). • Variation: While walking, the instructor randomly called out different directions (e.g., walking forward, backward, or sideways), speeds (slowly to quickly), pathways (e.g., walk in a straight, curved, or zigzag path), shapes (e.g., walk in the shape of a circle, a letter of the family name, or a number, such as a big or small “8”).	Joung et al. (7, 19), Gilbert (8)
Mirroring	• Two participants stand facing each other. One person becomes the leader and the other becomes the follower. The follower copies the leader's movements as accurately as possible. After a period of time, the pair switches roles so both participants experience leading and following.	Joung et al. (7, 19), Gilbert (8)
1, 2, 3 dance	• Participants choose three movements that they can perform (Movement 1, Movement 2, Movement 3). The instructor then calls out numbers in random order, and participants perform the corresponding movement to the rhythm of the music • Direction: “Follow the sequence when the numbers are called: (1, 2, 3), (2, 1, 3), (3, 2, 1), (1, 3, 2), …”.	Joung et al. (7)
Invisible ball	• Participants imagine that they are holding an invisible ball in their hand. They play with this imaginary ball by throwing it, catching it, or rolling it to another person. The ball's shape, weight, size, and texture can change freely according to their imagination. Participants are encouraged to use as much creativity as possible while engaging in this play.	Joung et al. (19)
Hand airplane	• The hand becomes an airplane that travels through space. The airplane may take off from the ground, fly into the air, shake up and down as if passing through a storm, or respond to various imagined situations. The instructor suggests a destination (e.g., Seoul to New York, Korea to Africa), which indicates how long participants should continue their movement.	Joung et al. (7)

Linking movement to imaginative frames is not unique to rehabilitation-oriented dance approaches. In dance/movement therapy (DMT), a clinical field that uses movement as a medium for symbolization, affective expression, and meaning-making, imagery and metaphor have long been employed to elicit and shape movement exploration across diverse populations, including individuals with intellectual disability, autism spectrum conditions, neurological disorders, and mood disorders (26, 27). However, DMT has traditionally emphasized expressive and psychosocial processes, whereas CBP in motor rehabilitation places a primary focus on functional movement practice and motor learning. In this context, imaginative framing in CBP can be understood as a pragmatic means of expanding the motor solution space and supporting engagement, rather than as an end in itself. Recent neurorehabilitation research has reported that imagery can activate motor-related neural systems and facilitate motor learning (28, 29). Dodakian et al. (30) reported that imagery-based tasks can enhance actual motor performance. Although these studies have primarily involved individuals post-stroke and further investigation is needed to determine whether similar effects extend to individuals with CP, incorporating imagery into motor training may offer practical insights for CP rehabilitation—particularly given the movement and motor-control difficulties commonly experienced by individuals with CP.

A critical consideration in interventions for individuals with CP is the need to support their motivation, attention, and self-efficacy, allowing them to experience a sense of “I can do this.” As Novak et al. (10) emphasize, motivation and attention are well-established modulators of intervention outcomes in CP, and successful task-specific practice tends to be rewarding and enjoyable, encouraging children to practice more spontaneously. Barnewolt (31) notes that reducing mental barriers in dance enables students with CP to “move with a freedom that had previously felt inaccessible,” highlighting the role of psychological safety in fostering engagement.

Qualitative studies further reveal how repeated failure experiences shape self-perception. Children report that difficulties in keeping up with peers or meeting task demands often lead to frustration, embarrassment, and withdrawal. As Lauruschkus et al. (32) observed, “children sometimes choose to step away from activities when expectations exceed their abilities.” Conchar et al. (33) likewise describe how shame related to “having a different body” can result in self-exclusion, and Shimmell et al. (34) note that fear of failure contributes to avoidance of challenging tasks.

Given this evidence, interventions must attend not only to physical performance but also to these intertwined psychological experiences. In this regard, CBP offers an open and accepting environment where individuals with CP can explore movement without pressure to perform “correctly.” By prioritizing generative and self-directed exploration, CBP supports the emergence of positive, embodied experiences that counteract prior patterns of failure, stigma, and diminished confidence.

Despite these strengths, the evidence base supporting CBP remains in its early stages, and several limitations should be considered when interpreting the findings of this review. The current evidence supporting CBP in CP is derived predominantly from pilot studies with small sample sizes (7, 18). These studies primarily included individuals classified as GMFCS level II who were capable of independent ambulation and were conducted mainly in adolescent or adult populations, leaving uncertainty regarding the applicability of these approaches to younger children or older adults with CP. Although randomized controlled trials by Teixeira-Machado et al. (4) and Teixeira-Machado and DeSantana (5) included individuals with more severe motor impairment who were not independently ambulatory (GMFCS levels III–V), the number of such studies remains limited. Therefore, the existing evidence base on CBP is not yet sufficient to establish a robust foundation for clinical application across the broader CP population.

A critical consideration in applying CBP to motor rehabilitation in CP is that CP is a highly heterogeneous condition. As is well recognized, CP is primarily characterized by motor impairment; however, individuals with CP exhibit heterogeneous motor features and functional levels depending on motor type (spastic, dyskinetic, ataxic, or mixed) and GMFCS levels. Moreover, comorbidities—including intellectual disability and other neurological or behavioral conditions—are common (35), which may influence task engagement, communication, and responsiveness to imagery- or narrative-based components. This wide spectrum may pose methodological challenges in achieving participant homogeneity and may limit generalization across subtypes and severity levels. It may give rise to practical constraints that are particularly salient for group-based dance interventions, such as safety monitoring, the need for assistants, and real-time task adaptation to accommodate mixed functional abilities. Accordingly, based on the evidence currently available in this review, it remains unclear under what conditions and for whom CBP optimally supports motor learning and engagement. Establishing a robust evidence base for CBP in CP will require future studies to employ well-designed clinical trials that compare CBP with established CP rehabilitation interventions, incorporate stratified samples accounting for CP type and GMFCS level, provide clear documentation of task adaptations, and systematically evaluate moderating variables.

### 3.4 Implications and future directions

CBP offers a structured yet flexible movement methodology that aligns with the core principles of modern CP rehabilitation—self-initiated practice, task relevance, variability, contextual adaptation, and motivational engagement—while introducing unique strengths derived from creative exploration, narrative framing, and expressive movement generation. By expanding motor solution spaces, enhancing salience and emotional investment, and integrating cognitive, emotional, and social dimensions into functional movement practice, CBP provides a promising and underexplored intervention framework for individuals with CP. López-Ortiz et al. (2) and Duarte Machado et al. (3) further emphasize that, for dance to be incorporated meaningfully within rehabilitation, dance-based interventions must include clear movement frameworks, standardized reporting of protocols, and rigorous evaluation of functional outcomes. Such efforts underscore the importance of detailed intervention reporting as a pragmatic alternative to rigid standardization when advancing dance-based practices within rehabilitation research. CBP likewise faces this methodological challenge. Although conceptually aligned with contemporary motor learning theory, it requires further well-designed trials and standardized documentation to establish its clinical utility, thereby indicating the need for a more systematic accumulation of evidence before dance-based, movement-centered practices can be confidently integrated into CP rehabilitation.
